# Sex-Based Differences in the Physical Capacity Profile of Regional Fencers

**DOI:** 10.3390/sports14060238

**Published:** 2026-06-09

**Authors:** Javier Gaviria Chavarro, Óscar Hernán Jiménez Trujillo, Miguel Ángel Gómez García, Rosa Nury Zambrano Bermeo, Catalina Jiménez Cerquera

**Affiliations:** 1 Doctoral Program in Applied Sciences, Faculty of Basic Sciences, Universidad Santiago de Cali, Cali 760011, Colombia; 2 Faculty of Sport and Education Sciences, Institución Universitaria Escuela Nacional del Deporte, Cali 760033, Colombia; oscar.jimenez@endeporte.edu.co (Ó.H.J.T.); mangelgomez@endeporte.edu.co (M.Á.G.G.); 3 Nursing Program, Faculty of Health, Universidad Santiago de Cali, Cali 760011, Colombia; rosa.zambrano00@usc.edu.co; 4 Physiotherapy Program, Faculty of Health, Universidad Santiago de Cali, Cali 760011, Colombia; catalina.jimenez00@usc.edu.co

**Keywords:** athletic performance, cardiorespiratory fitness, causality, sex differences, physical fitness

## Abstract

Fencing is an intermittent combat sport in which performance depends on the interaction of neuromuscular qualities, aerobic support, and weapon-specific demands. However, evidence on sex-based differences in the physical capacity profiles of regional fencers remains limited. This study compared the physical capacity profiles of 27 fencers from the Liga Vallecaucana de Esgrima (13 women and 14 men; 14–31 years) in an observational, cross-sectional, comparative study. Field-based assessments included push-ups, sit-ups, squats, jump squats, pull-ups, terminal speed attained in the 20-m shuttle run test, and estimated VO2max. The analysis adopted an exploratory, estimation-oriented approach based on mean differences, 95% confidence intervals, Hedges’ g, supplementary significance testing, false discovery rate adjustment, and a directed acyclic graph to clarify causal assumptions. The most robust sex-based difference was observed in pull-up performance, with men outperforming women by 5.43 repetitions (95% CI: 3.51 to 7.45; g = 1.88), and this was the only comparison retained after FDR correction. No conclusive sex-based differences were found for push-ups, sit-ups, squats, jump squats, terminal shuttle-run speed, or estimated VO2max. Mean estimated VO2max for the overall sample was 43.48 ± 9.12 mL·kg^−1^·min^−1^. These findings suggest that upper-limb pulling strength may be the main distinguishing physical quality in this cohort, although its implications for individualized conditioning remain to be established. Nevertheless, the results should be interpreted as observational associations rather than causal effects because of the cross-sectional design, the small sample, the field-based measurements, the imbalance in weapon distribution, and the lack of standardized measures of training exposure.

## 1. Introduction

Fencing is an Olympic combat sport characterized by brief, high-intensity actions, substantial neuromuscular demands, and a considerable cognitive-perceptual load. During a bout, athletes alternate between offensive actions, defensive maneuvers, feints, and counterattacks, while also executing rapid changes in pace and direction, together with short, explosive footwork and weapon-specific movements such as the lunge. This intermittent nature of performance requires technical precision under fatigue, postural control, and the capacity to repeatedly produce explosive efforts with minimal deterioration in technical execution [1]. In addition, the physiological and physical fitness profile of fencing reflects a reliance on the aerobic system, which is essential for rapid recovery between bouts. Previous studies have also shown that VO2max varies according to weapon category, with épée fencers generally demonstrating higher values than sabre and foil fencers [2,3,4]. Similarly, the distribution of exercise intensity during a round of bouts has been described, with approximately 40–45% of efforts performed at moderate intensity and 5–10% at high intensity, while the remaining proportion is distributed across low or light intensities [3,5].

In this context, physical preparation plays a fundamental role. Lower-limb strength and power contribute to postural control, advance–retreat speed, and lunge effectiveness; upper-limb strength may support weapon stability and force transmission during offensive actions; and aerobic capacity facilitates recovery between actions and bouts during prolonged competitive events [3,6,7,8]. Given that athletes maintain specific combat stances, rely on a dominant arm, and often prefer particular grip types, individualized training becomes especially important. Accordingly, training programs should tailor exercise selection and training load to these technical and sport-specific characteristics [8,9,10].

Sex-related differences in athletic performance have been consistently documented across a wide range of sporting contexts; however, their magnitude and practical expression are not uniform and depend on developmental stage, training background, and event-specific demands. Puberty is generally regarded as the period in which performance trajectories begin to diverge more clearly, largely because the marked increase in circulating testosterone in males coincides with substantial gains in muscle mass, strength, and power, as well as with changes that may influence specific components of endurance performance [11,12,13]. In this context, the American College of Sports Medicine has identified greater skeletal muscle mass, a higher proportion of type II muscle fibers, and larger muscle cross-sectional area as key physiological factors that may contribute to male advantage in strength- and power-oriented tasks. By contrast, females generally exhibit a greater relative proportion of type I fibers and a more favorable oxidative profile, characteristics that may be beneficial in tasks involving prolonged submaximal effort or enhanced fatigue resistance [12].

Cardiovascular differences have also been proposed as relevant contributors to sex-related variation in physical performance. Males typically present with larger cardiac dimensions, greater stroke volume, and higher hemoglobin concentrations, all of which support a greater capacity for oxygen delivery during exercise. Females, in contrast, generally exhibit lower maximal aerobic capacity, partly associated with smaller cardiac and pulmonary dimensions. Nevertheless, interpreting these differences as a universal disadvantage for women would be overly simplistic, as evidence from endurance and ultra-endurance contexts indicates that women may perform comparably to or even outperform men under specific competitive and physiological conditions [12,13].

Anthropometric characteristics provide an additional explanatory framework. On average, males tend to be taller, possess longer limbs, and exhibit greater fat-free mass, attributes that may enhance torque generation and absolute force production. Females, conversely, tend to present with shorter limb lengths and a higher relative fat mass. While these morphological differences are often used to explain disparities in mechanical output and physical performance, such interpretations should be made with caution, since athletic performance cannot be reduced to isolated anatomical or physiological traits, but instead emerges from the interaction of morphology, endocrine factors, neuromuscular characteristics, training exposure, technical proficiency, and task-specific constraints [13].

In fencing, sex-related differences remain comparatively underexplored, and clear physiological and anthropometric distinctions across athlete profiles or weapon-specific modalities have yet to be systematically established. Nonetheless, previous evidence indicates that women exhibit approximately 40–50% lower upper-limb strength and 20–40% lower lower-limb strength than men [12]. Within fencing, sex-based comparisons have also been discussed in relation to elite performance and weapon-specific contexts, although the evidence remains limited [11]. Therefore, it may be argued that male fencers could retain certain performance advantages in contexts where force production contributes meaningfully to execution. However, such an assumption should be advanced with caution, as fencing performance is inherently multifactorial and cannot be inferred from strength differentials alone. Rather, it emerges from the interaction of physical capacities with technical proficiency, tactical adaptability, neuromuscular coordination, and the specific demands imposed by each weapon and competitive context [7,8].

Modern training increasingly emphasizes individualized approaches that acknowledge biological heterogeneity and differences in training volume and load exposure. In fencing, this perspective is relevant because physical preparation must be aligned with technical, anthropometric, weapon-specific, and performance-related demands [6,8,10]. In fencing research, the current trend likewise points toward a more specific and tailored approach in which athletes’ technical, anthropometric, and other sport-relevant characteristics are considered in the planning process [10]. Although some studies have examined the anthropometric and physiological characteristics of elite and sub-elite fencers, important gaps remain in the comparative characterization of regional athletes from a sex-based perspective. This limitation restricts the direct transfer of findings from elite or international samples to local athlete development contexts [6,10,11].

A substantial theoretical gap remains regarding physical capacity profiles that account for sex-based differences in fencers. There is currently limited evidence on physiological differences across weapon modalities, and available information on sex-based differences and their influence on fencing performance remains insufficient [2,4,10,11]. This lack of analysis from a sex-based perspective represents an important opportunity for innovation and for the development of new approaches to fencing and its training. Accordingly, further research in this area is warranted.

Additionally, in studies with multiple outcomes, reliance on statistical significance alone may inflate the probability of false-positive findings and obscure the magnitude of observed differences; therefore, false discovery rate procedures can help control multiplicity-related error [14]. Hypothesis tests should also be complemented with effect sizes and confidence intervals, and analytical assumptions should be made explicit through causal frameworks such as directed acyclic graphs (DAGs) [15,16,17]. On this basis, the aim of the present study was to analyze sex-based differences in the physical capacity profile of fencers from the Liga Vallecaucana de Esgrima.

## 2. Materials and Methods

An observational, cross-sectional study with a descriptive and comparative analytical focus was conducted to characterize the physical capacity profile of fencers affiliated with the Valle del Cauca Fencing League and to examine sex-based differences based on a single field-based assessment session [18,19,20].

The study was carried out at the Valle del Cauca Fencing League in Cali, Colombia. The target population comprised all active fencers affiliated with the League during the assessment period. A total of 27 athletes were evaluated, including 13 women and 14 men, aged 14 to 31 years (overall mean: 18.07 ± 4.14 years). Rather than a selected sample, the study included the entire accessible population of active League athletes who were available and eligible to be assessed at that time. This allowed a direct characterization of that specific setting, although the findings should not be generalized automatically to fencers from other leagues, regions, or competitive levels [18,19,20].

Because all accessible active athletes were included during the study period, no a priori sample size calculation was used to define recruitment targets. The study was therefore approached as exploratory. Nevertheless, the total number of participants was small, which limited estimation precision and reduced the ability to detect small or moderate sex-based differences. For this reason, interpretation was based not only on *p* values but also on effect magnitudes and confidence intervals.

The variables analyzed were age, sex, weapon category, and performance in field-based tests intended to reflect muscular strength/endurance and aerobic fitness. Recorded measures included push-ups, sit-ups, squats, jump squats, pull-ups, terminal speed attained in the 20-m shuttle run test, and estimated VO2max [21]. Sex was treated as the main grouping variable for the comparisons, whereas age and weapon category were regarded as relevant contextual variables for interpreting the observed performance pattern. Because the athletes covered a broad age range, age was considered a potential source of biological and training-related heterogeneity, although the group size did not support more complex analytical structures for all variables analyzed [22,23,24].

Weapon category was available for all athletes and is reported descriptively by sex. According to coaching records, years of fencing practice in the evaluated cohort ranged from 3 to 15 years. However, individual-level experience values were not consistently available for tabulation by sex in the study database. Likewise, competitive level, recent injury status, and systematic weekly training volume were not recorded in a sufficiently standardized manner to support formal descriptive tabulation or comparative analysis. These omissions should be considered when interpreting between-sex comparisons.

The assessment battery consisted of field tests selected to represent physical components of interest in fencing, particularly muscular strength/endurance and aerobic fitness. Muscular strength/endurance was assessed through push-ups performed to failure, sit-ups completed in 2 min, squats completed in 1 min, jump squats completed in 1 min, and pull-ups performed to failure. Although these tests do not reproduce all technical and tactical demands of fencing bouts, they provide a practical approximation of the athlete’s general neuromuscular profile and a useful baseline for training planning in applied sport settings. Accordingly, the battery should be interpreted as a general physical profile rather than as a direct measure of fencing-specific performance.

Field-based tests were selected because they are feasible, low-cost, and commonly used in applied sport settings to characterize general components of physical fitness when laboratory-based assessment is not available [25]. Nevertheless, their interpretation was restricted to general physical capacity rather than fencing-specific performance. Previous evidence indicates that body-weight muscular fitness tests can provide acceptable validity and reliability for group-level assessment of strength-endurance capacities in physically active or healthy young adult populations, although their interpretation may vary according to protocol standardization, scoring method, movement criteria, participant age, and the specific construct being measured [26,27]. Therefore, the push-up, sit-up, squat, jump-squat, and pull-up tests were interpreted as practical indicators of upper-limb, trunk, and lower-limb muscular endurance rather than as isolated measures of maximal strength or direct determinants of fencing performance. In contrast, the 20-m shuttle run test has been widely used as a practical field-based indicator of cardiorespiratory fitness, with evidence supporting its feasibility, reliability, and criterion-related validity for estimating aerobic fitness at the group level, although estimated VO2max should not be interpreted as a direct physiological measurement [28,29,30]. These considerations support the use of the battery for applied physical profiling, while requiring cautious interpretation in a heterogeneous sample of regional fencers because fencing performance involves intermittent actions, short displacements, lunges, changes in direction, and perceptual-response demands that are not fully reproduced by general field tests [1,7,8].

Athletes were familiarized with the procedures before formal recording. The strength/endurance tests were administered in blocks of approximately five athletes so that recovery could occur while other athletes completed their trials. All blocks followed the same operational order during each testing day, and one valid score per participant was recorded for each test according to the applied field protocol. For the strength/endurance battery, the recorded score corresponded to the maximum number of valid repetitions completed under the specific rule assigned to each test: push-ups and pull-ups were performed to failure, squats and jump squats were counted over 1 min, and sit-ups were counted over 2 min. The 20-m shuttle run was administered on a separate day to avoid excessive accumulated fatigue and to preserve adequate recovery before the aerobic assessment.

Aerobic fitness was assessed with the 20-m shuttle run test, from which both terminal shuttle-run speed and an indirect estimate of VO2max were obtained. This test was selected because of its operational feasibility in a real-world group assessment context where direct ergospirometric measurement was not available. Accordingly, a standardized and easily implemented field-based procedure was prioritized, while recognizing that the resulting VO2max value represents an estimate rather than a direct physiological measurement of maximal oxygen uptake [21,28,29,30,31].

In this study, estimated VO2max was calculated using the equation VO2max = 31.025 + 3.238V − 3.248E + 0.1536(V × E), where V denotes the terminal speed reached during the shuttle-run protocol, expressed in km/h, and E denotes age in years. One record was obtained for each participant from the test protocol. Because terminal shuttle-run speed forms part of the estimation procedure for VO2max, both variables should be interpreted as complementary indicators derived from the same incremental field protocol rather than as fully independent physiological outcomes. Terminal speed reflects performance attained within the test itself, whereas estimated VO2max is a derived cardiorespiratory index adjusted for age according to the shuttle-run estimation model [30].

Assessments were conducted over a one-week period under standardized conditions and under the supervision of trained evaluators. Before test administration, participants completed a standardized warm-up to reduce variability related to immediate preparation and to promote safe execution of the full testing battery. Results were recorded immediately after each assessment, coded to protect participant identity, and stored exclusively for academic and research purposes.

Because the study was conducted in an applied rather than a laboratory setting, the tests should be interpreted as tools for functional characterization rather than as instruments intended to isolate specific physiological mechanisms experimentally. In this sense, the battery provides a practical approximation of the fencer’s general physical profile and may offer useful contextual information for training planning in applied settings, while remaining subject to the limitations inherent to field testing [25,26,27].

Given the observational cross-sectional design, comparisons between women and men were interpreted as observed associations between groups rather than as direct causal effects. To make the underlying analytical assumptions explicit and to delimit the inferential scope more clearly, a directed acyclic graph (DAG) was developed for pull-up performance, taking sex as the exposure of interest [15,16,17]. Age and weapon category were incorporated as observed variables, whereas years of experience and training load were conceptualized as unmeasured variables plausibly related to performance and to group comparability [15,16,17]. Under this conceptual structure, no valid adjustment set was identified with the available information for estimating the total causal effect of sex on pull-up performance. Therefore, pull-up findings were interpreted from a strictly descriptive and comparative perspective, explicitly acknowledging residual confounding and the limitations arising from the asymmetric distribution of weapon category, particularly the absence of women in épée.

### 2.1. Statistical Analysis

Variables were first summarized using means and standard deviations for the overall sample and by sex. Approximate normality within each group was then assessed using the Shapiro–Wilk test. Although analytical decisions were not based exclusively on this test, it served as a preliminary examination of distributional behavior in a small-to-moderate dataset.

The inferential framework was exploratory and estimation oriented. For all analyzed variables, primary emphasis was placed on mean differences, 95% confidence intervals, and standardized effect sizes estimated with Hedges’ g. For comparisons considered compatible with a parametric approach, 95% confidence intervals for the mean difference were calculated using Welch’s method. For comparisons in which a non-parametric approach was preferred, 95% confidence intervals for the mean difference were obtained by bootstrap resampling. Null-hypothesis tests were reported as supplementary, distribution-informed complements rather than as the principal basis for interpretation.

Sex-based comparisons were therefore complemented with Welch’s *t*-test when a parametric approach was considered acceptable and with the Mann-Whitney U test when that condition was not sufficiently supported. In this context, Mann-Whitney U results should be interpreted as ancillary non-parametric evidence, whereas the reported mean difference and its confidence interval remained the main estimation target.

Because seven analyzed variables were compared, false discovery rate control was applied using the Benjamini-Hochberg procedure with alpha set at 0.05 [14]. This strategy was intended to reduce the probability of spurious findings arising from multiple comparisons without imposing an excessively conservative penalty on analytical sensitivity. All analyses were performed in Python(version 3.12) using libraries for data handling, numerical computation, and statistical analysis, including pandas, numpy, scipy, and statsmodels.

### 2.2. Ethical Considerations

The study was conducted in accordance with the ethical principles of the Declaration of Helsinki for research involving human participants. Written informed consent was obtained from all adult participants. For minors, written informed consent was obtained from parents or legal guardians, and assent was obtained from the participating minors. Confidentiality was ensured through coded records, and the data was used exclusively for academic and research purposes.

## 3. Results

A total of 27 fencers from the Valle del Cauca Fencing League were included, comprising 13 women and 14 men. The mean age of the overall sample was 18.07 ± 4.14 years.

Table 1 presents the descriptive characteristics of the participants. Weapon category differed across sexes, with no women represented in epee. Foil was the most frequent weapon among women, whereas men were distributed across all three weapon categories. This imbalance should be considered when interpreting the between-sex comparisons reported below.

Across all participants, push-up performance ranged from 1 to 60 repetitions (27.22 ± 11.23), sit-up performance from 29 to 92 repetitions (61.74 ± 16.19), jump squats from 35 to 77 repetitions (53.07 ± 11.62), squats from 41 to 90 repetitions (59.22 ± 13.46), and pull-ups from 1 to 15 repetitions (4.81 ± 3.89). Terminal shuttle-run speed ranged from 10.0 to 12.5 km/h (11.43 ± 0.66), whereas estimated VO2max ranged from 24.11 to 53.75 mL·kg^−1^·min^−1^ (43.48 ± 9.12).

Table 2 summarizes the descriptive performance statistics by sex. Overall, most variables showed either small descriptive differences or moderate contrasts accompanied by substantial uncertainty. The clearest descriptive contrast was observed for pull-up performance, with higher values in men than in women (7.43 ± 3.65 vs. 2.00 ± 1.35 repetitions). More moderate descriptive differences were also observed for terminal shuttle-run speed and estimated VO2max, although these estimates remained subject to considerable uncertainty.

Table 3 presents the exploratory sex-based comparisons using an estimation-oriented framework, including mean differences between men and women, 95% confidence intervals, standardized effect sizes, unadjusted *p* values, and false discovery rate adjustment. The most robust finding corresponded to pull-up performance, for which men outperformed women by 5.43 repetitions (95% CI: 3.51 to 7.45; Hedges’ g = 1.88). This was the only analyzed variable that remained statistically compatible with a between-sex difference after false discovery rate adjustment (FDR-adjusted *p* < 0.001).

For the remaining variables, no conclusive between-sex differences were observed after multiplicity control. Push-ups showed a mean difference favoring women (Δ[M-F] = −5.21 repetitions; 95% CI: −12.97 to 2.88; g = −0.45). Sit-ups and jump squats displayed small mean differences and trivial-to-small standardized effects, with confidence intervals that overlapped the null. A similar pattern was observed for squats, in which the contrast slightly favored men but remained imprecise.

Terminal shuttle-run speed showed a mean difference of 0.47 km/h in favor of men (95% CI: −0.02 to 0.97; g = 0.74). Although the standardized difference was moderate, the interval estimates still included values compatible with little or no difference, and the FDR-adjusted *p* value did not support a conclusive between-sex contrast (0.210). Estimated VO2max differed by 2.80 mL·kg^−1^·min^−1^ in favor of men (95% CI: −3.95 to 9.36; g = 0.30), with substantial uncertainty and no robust inferential support after multiplicity adjustment (FDR-adjusted *p* = 0.663). Taken together, these findings indicate that, within this sample, pull-up performance was the only analyzed variable showing a clear and statistically robust sex-based difference.

**Figure 1:**
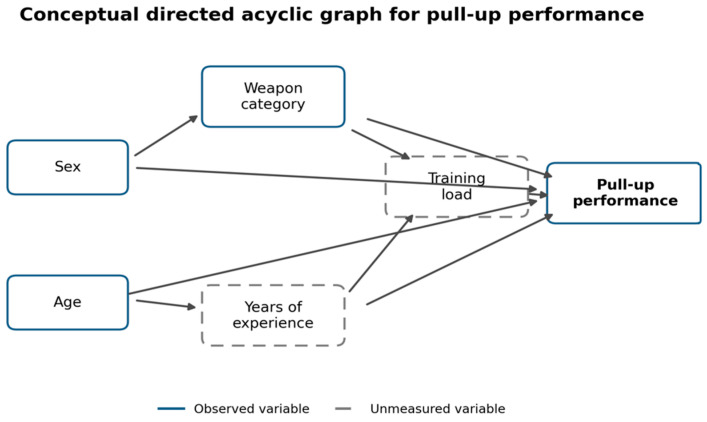
Conceptual directed acyclic graph for pull-up performance. **Figure note.** Observed variables are shown with solid borders, whereas unmeasured variables are shown with dashed borders. The graph makes explicit the assumed relationships among sex, age, weapon category, years of experience, training load, and pull-up performance. Under this structure, no valid adjustment set was identified with the available information for estimating the total causal effect of sex on pull-up performance, supporting a strictly descriptive and comparative interpretation of the observed group differences.

**Figure 2:**
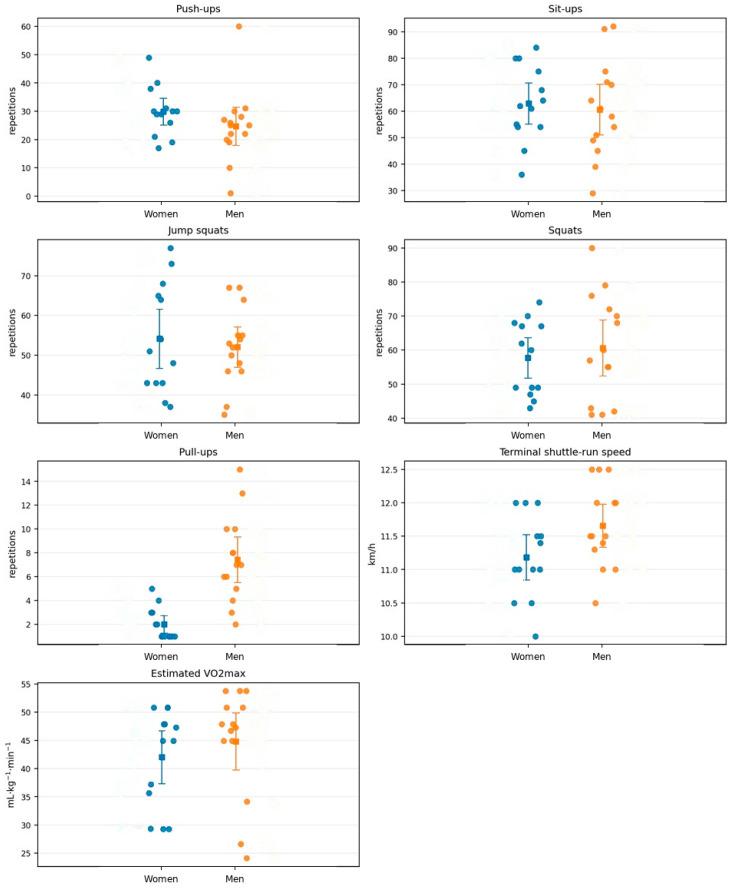
Raw outcome distributions by sex. **Figure note.** Each point represents one participant. Squares and error bars indicate the group mean and its approximate 95% confidence interval. This figure is provided to facilitate direct inspection of dispersion, overlap between groups, and potential floor or ceiling effects across outcomes.

Figure 1 presents the conceptual DAG used to make explicit the assumed relationships among sex, age, weapon category, years of experience, training load, and pull-up performance. Under this structure, no valid adjustment set was identified with the available information for estimating the total causal effect of sex on pull-up performance, supporting a strictly descriptive and comparative interpretation of the observed group differences. Figure 2 displays the raw outcome distributions by sex, allowing direct inspection of dispersion, overlap, and potential floor effects across variables.

## 4. Discussion

The main finding of this study was a clear sex-based difference in pull-up performance, with men achieving higher scores than women. This difference was large in magnitude (Hedges’ g = 1.88), corresponded to a mean difference of 5.43 repetitions, and remained statistically robust after FDR adjustment. In practical terms, upper-limb pulling strength appeared to be the most distinctive physical capacity between sexes in this cohort. However, this result should be interpreted cautiously, as the present study did not assess fencing-specific performance outcomes and therefore cannot determine whether higher pull-up performance translates into improved competitive performance [7,8,32,33].

In contrast, no conclusive sex-based differences were observed for push-ups, sit-ups, squats, jump squats, terminal shuttle-run speed, or estimated VO2max after adjustment for multiple comparisons. This finding should not be interpreted as evidence of equivalence between sexes. Given the exploratory design and small sample size, the confidence intervals remained compatible with small-to-moderate effects in either direction. Therefore, the combined interpretation of mean differences, confidence intervals, and standardized effect sizes provides a more informative basis for interpretation than statistical significance alone.

For terminal shuttle-run speed, men showed a moderate descriptive advantage, but the confidence interval included values compatible with little or no difference, and the comparison did not remain significant after FDR adjustment. Because terminal speed in the 20-m shuttle run reflects incremental running performance, it should not be interpreted as a direct measure of fencing-specific speed. This distinction is important because fencing involves short accelerations, decelerations, changes in direction, lunges, and responses to external stimuli [1,3,7,8,34,35]. Future studies should include more specific assessments, such as lunge time, short multidirectional displacements, and reactive agility tasks, using protocols designed for fencing performance [34,35,36,37,38].

Estimated aerobic capacity was broadly consistent with the intermittent demands of fencing, in which aerobic fitness supports recovery between high-intensity actions and bouts [1,2,3,4,5]. However, because VO2max was estimated from the 20-m shuttle run rather than measured directly, the values should be interpreted as field-based approximations rather than direct physiological measurements [21,28,29,30,31]. The small and non-conclusive sex-based difference suggests that aerobic conditioning remains relevant for both groups. Nevertheless, the present data do not support weapon-specific aerobic prescriptions, since the analysis was not stratified by weapon and did not evaluate training responses. Training recommendations based on high-intensity intervals, plyometric work, strength-oriented conditioning, or weapon-specific work-to-rest ratios should therefore be considered literature-based programming references rather than direct implications of the present findings [38,39,40,41].

The conceptual DAG helped clarify the inferential limits of the study. Weapon distribution differed by sex, with no women represented in épée, and relevant variables such as fencing experience and training load were not measured systematically. Consequently, the observed differences should be interpreted as descriptive associations rather than causal effects [15,16,17]. This is particularly important for pull-up performance, where the large observed difference may still be influenced by residual confounding and limited overlap between groups.

The broad age range of the sample also introduced uncertainty. Participants were between 14 and 31 years old, which likely increased developmental and training-related heterogeneity. In athletic populations, chronological age does not necessarily reflect biological maturation, and maturation-related differences may affect body size, muscle mass, strength, power, and aerobic performance, especially during adolescence and early adulthood [22,23,24]. Therefore, the inclusion of adolescent and adult athletes in the same comparisons may have increased within-group variability and reduced comparability between sexes. Future studies should use narrower age bands or include maturation-related indicators, training history, and cumulative training exposure in the analytical framework.

### Limitations and Future Research

The main limitations of this study should be considered when interpreting the findings. First, the cross-sectional design precludes causal inference and does not capture longitudinal adaptations [15,16,17,20]. Second, the sample was small, which reduced precision and limited the ability to detect small effects. Third, potentially relevant contextual variables—including years of practice, weekly training volume, competitive level, and recent injury status—were not recorded in a sufficiently standardized manner. Fourth, weapon distribution was imbalanced across sexes, particularly because no women were represented in épée. Fifth, the assessment battery relied on field tests that provide a practical functional profile but do not isolate fencing-specific performance and are subject to measurement error, day-to-day variability, and the limitations inherent to the indirect estimation of aerobic capacity from the 20-m shuttle run test [21,25,26,27,28,29,30,31]. Future research should adopt longitudinal or interventional designs, include better characterization of training exposure, balance samples by sex and weapon or stratify analyses accordingly, and incorporate more sport-specific biomechanical and performance measures [7,8,34,35,36,37,38].

## 5. Conclusions

Among fencers from the Liga Vallecaucana de Esgrima, the most consistent sex-based difference was observed in pull-up performance, suggesting a relevant between-sex contrast in upper-limb pulling strength within this cohort. The other physical capacities assessed showed no conclusive sex-based differences after FDR control, although small effects cannot be ruled out. These findings may inform individualized training considerations, particularly regarding upper-limb pulling strength, although such implications should be interpreted cautiously given the observational design and the absence of direct fencing-performance outcomes. The results should be interpreted as observational associations, given the imbalance in weapon distribution and the lack of standardized measures of training exposure.

## Figures and Tables

**Table 1:** Participant characteristics by sex.

Characteristic	Total	Women (*n* = 13)	Men (*n* = 14)
Age (years), mean ± SD	18.07 ± 4.14	17.54 ± 2.76	18.57 ± 5.17
**Weapon category, *n* (%)**			
Épée	4 (14.8)	0 (0.0)	4 (28.6)
Foil	13 (48.1)	8 (61.5)	5 (35.7)
Sabre	10 (37.0)	5 (38.5)	5 (35.7)

Note. Percentages for weapon category are column percentages within sex and overall percentages for the total sample. According to coaching records, years of fencing practice ranged from 3 to 15 years across the cohort, although individual-level values were not consistently available for tabulation by sex. Competitive level, recent injury status, and systematic weekly training volume were not available in a sufficiently standardized format for inclusion in the descriptive table.

**Table 2:** Descriptive performance statistics (mean ± SD) by sex.

Variable	Total	Women (*n* = 13)	Men (*n* = 14)
Push-ups (repetitions)	27.22 ± 11.23	29.92 ± 8.73	24.71 ± 12.95
Sit-ups (repetitions)	61.74 ± 16.19	62.92 ± 14.36	60.64 ± 18.21
Jump squats (repetitions)	53.07 ± 11.62	54.15 ± 13.72	52.07 ± 9.70
Squats (repetitions)	59.22 ± 13.46	57.69 ± 10.95	60.64 ± 15.72
Pull-ups (repetitions)	4.81 ± 3.89	2.00 ± 1.35	7.43 ± 3.65
Terminal shuttle-run speed (km/h)	11.43 ± 0.66	11.18 ± 0.63	11.66 ± 0.62
Estimated VO2max (mL·kg^−1^·min^−1^)	43.48 ± 9.12	42.03 ± 8.64	44.83 ± 9.66

Note. Terminal shuttle-run speed refers to the final speed attained during the 20-m shuttle run protocol.

**Table 3:** Exploratory sex-based comparisons: mean difference, 95% confidence interval, effect size, and false discovery rate control.

Variable	Ancillary Test	Δ (M-F)	95% CI	Hedges’ g	*p*	FDR-Adjusted *p*
Push-ups (repetitions)	Mann-Whitney U test	−5.21	[−12.97, 2.88]	−0.45	0.103	0.241
Sit-ups (repetitions)	Welch’s *t*-test	−2.28	[−15.25, 10.69]	−0.13	0.720	0.720
Jump squats (repetitions)	Welch’s *t*-test	−2.08	[−11.65, 7.48]	−0.17	0.656	0.720
Squats (repetitions)	Welch’s *t*-test	2.95	[−7.77, 13.67]	0.21	0.575	0.720
Pull-ups (repetitions)	Mann-Whitney U test	5.43	[3.51, 7.45]	1.88	<0.001	<0.001
Terminal shuttle-run speed (km/h)	Welch’s *t*-test	0.47	[−0.02, 0.97]	0.74	0.060	0.210
Estimated VO2max (mL·kg^−1^·min^−1^)	Mann-Whitney U test	2.80	[−3.95, 9.36]	0.30	0.379	0.663

Note. Positive Δ (M-F) values indicate higher scores in men. For terminal shuttle-run speed, positive values indicate higher terminal speed in men. The study was interpreted using an estimation-oriented exploratory framework, with mean differences, confidence intervals, and Hedges’ g treated as the primary summaries. Welch’s *t*-tests and Mann-Whitney U tests were reported as ancillary distribution-informed complements. For Welch’s *t*-tests, the 95% confidence interval was calculated using Welch’s method; for Mann-Whitney U comparisons, the 95% confidence interval was obtained by bootstrap resampling for the mean difference. False discovery rate adjustment was applied across the seven analyzed variables.

## Data Availability

The datasets generated and/or analyzed during the current study are not publicly available due to ethical and privacy considerations but are available from the corresponding author upon reasonable request.

## Abbreviations

The following abbreviations are used in this manuscript:

DAG	Directed Acyclic Graph
VO2max	Maximum Oxygen Consumption
FDR	False Discovery Rate
SD	Standard Deviation
CI	Confidence Interval
